# Risk perception related to COVID-19 among the Iranian general population: an application of the extended parallel process model

**DOI:** 10.1186/s12889-020-09681-7

**Published:** 2020-10-19

**Authors:** Leila Jahangiry, Fatemeh Bakhtari, Zahara Sohrabi, Parvin Reihani, Sirous Samei, Koen Ponnet, Ali Montazeri

**Affiliations:** 1 Tabriz Health Services Management Research Center, Tabriz University of Medical Sciences, Tabriz, Iran; 2 Health Education and Health Promotion Department, School of Public Health, Tabriz University of Medical Sciences, Tabriz, Iran; 3 Medical Education Research Center, Health Management and Safety Promotion Research Institute, Tabriz University of Medical Sciences, Tabriz, Iran; 4 Faculty of Social Sciences, Imec-mict Ghent University, Ghent, Belgium; 5 Population Health Research Group, Health Metrics Research Center, Institute for Health Sciences Research, ACECR, Tehran, Iran; 6 Faculty of Humanity Sciences, University of Science and Culture, Tehran, Iran

**Keywords:** Extended parallel process model, Coronavirus disease 2019, Fear control, Danger control, Behavior response

## Abstract

**Background:**

The novel coronavirus disease 2019 (COVID-19) has emerged as a major global public health challenge. This study aimed to investigate on how people perceive the COVID-19 outbreak using the components of the Extended Parallel Process Model (EPPM) and to find out how this might contribute to possible behavioral responses to the prevention and control of the disease.

**Methods:**

This cross-sectional study was conducted in Iran during March and April 2020. Participants were recruited via online applications using a number of platforms such as Telegram, WhatsApp, and Instagram asking people to take part in the study. To collect data an electronic self-designed questionnaire based on the EPPM was used in order to measure the risk perception (efficacy, defensive responses, perceived threat) related to the COVID-19. Descriptive statistics, chi-square, t-test and analysis of variance (ANOVA), were used to explore the data.

**Results:**

A total of 3727 individuals with a mean age (SD) of 37.0 (11.1) years participated in the study. The results revealed significant differences in efficacy, defensive responses and perceived threat among different population groups particularly among those aged 60 and over. Women had significantly higher scores than men on some aspects such as self-efficacy, reactance, and avoidance but men had higher perceived susceptibility scores compared to women. Overall 56.4% of participants were engaged in danger control (preventive behavior) while the remaining 43.6% were engaged in fear control (non-preventive behavior) process.

**Conclusion:**

More than half of all participants motivated by danger control. This indicated that more than half of participants had high perceived efficacy (i.e., self-efficacy and response efficacy). Self-efficacy scores were significantly higher among participants who were older, female, single, lived in rural areas, and had good economic status. The results suggest that socioeconomic and demographic factors are the main determinants of the COVID-19 risk perception. Indeed, targeted interventions are essential for controlling the pandemic.

**Supplementary information:**

**Supplementary information** accompanies this paper at 10.1186/s12889-020-09681-7.

## Background

The novel coronavirus disease 2019 (COVID-19) has emerged as a major global public health challenge [1]. This potentially fatal infectious disease, which has affected most countries worldwide, is characterized by a steady speed of spread, leading to the World Health Organization (WHO) classifying it as a pandemic [2]. In Iran, apparently the two first cases of deaths related to COVID-19 were reported on February 19, 2020 from Qom, a city in the central part of the country. As of April 13, 73,303 Iranian people have been infected with the COVID-19. In total, 4585 have died and 45,983 have recovered when we were carrying this study [3].

The COVID-19 is transmitted from human to human through respiratory droplets or direct contact. Available findings show that avoiding exposure is the key to preventing COVID-19 infection, which is why quarantining, physical distancing, and isolation have become the primary strategies for reducing COVID-19 incidence and mortality. Quarantine restricts the movement of people and reduces the infection rate for contagious diseases [4]; physical or social distancing involves staying at least two meters away from other persons; and isolation is the state of separating patients with COVID-19 from otherwise healthy people [5]. Maintaining hygiene practices, such as proper hand washing, wearing a mask in crowded places, and staying at home, all are recommended for controlling the spread of the disease and breaking the transmission chain [6].

During the early stages of the coronavirus pandemic in Iran, several social media campaigns were launched to inform people about the risks of coronavirus and to persuade them to follow health care recommendations. Therefore, providing necessary information about people’s perception related to COVID-19 is important for health policy makers in implementing effective and appropriate strategies in order to prevent and control of the disease.

There are several factors that might affecting COVID-19 response behaviors [7]. Multiple health models suggest that risk perception of the COVID-19 is a vital component for any behavior change [8]. Of these, the extended parallel process model (EPPM) is very relevant [9]. According to the EPPM, when people are exposed to a risky situation, they go through two cognitive appraisals: one related to the efficacy of the recommended advice and one related to the perceived threat [10]. The EPPM therefore indicates that the perception of a risk depends on efficacy, defensive response and perceived threat [11]. Thus, in order to influence people’s behavior to follow the COVID-10 health recommendations it is important to understand how people perceive the COVID-19 pandemic, how they are assessing these risks, and how such assessments might lead them to change their behaviors. This study aimed to investigate on how people have perceived the COVID-19 outbreak using the components of the EPPM (efficacy, defensive responses, perceived threat) and how these might contribute to possible behavioral responses to the prevention and control of the disease.

## Methods

### Design

This was an online cross sectional study that was carried out in Iran during March to April 2020.

### Data collection and participants

Data were collected using an electronic questionnaire via Porsline. Participants were recruited using online applications and posts on several platforms such as Telegram, WhatsApp, and Instagram. We asked people for sharing the study announcements and to participate in the study. We also send several messages to significant others, virtual groups to share the study announcements. The posts asked people aged 15 years and over to take part in a study that investigates on the COVID-19. Those interested in participating were directed to complete the online questionnaire.

### Sample size estimation

The sample size for the study was estimated using the following formula [12]:

$$ \mathrm{n}={\mathrm{Z}}^2\mathrm{P}\left(1-\mathrm{P}\right)/{\mathrm{d}}^2 $$

where Z for 95% confidence interval is 1.96, *P* = 0.5 (assuming that 50% of people would either be engaged in danger control or fear control processes), and d = 0.02 (precision = 2%). As such we estimated at least 2400 participants would be requiring for the study to have a power of 80% at 5% significant level. However, in practice 3727 individuals participated in the study.

### The study questionnaire

The Risk-Percept COVID-19 was used to collect the data. This questionnaire was developed based on a literature review of the EPPM-based risk perception assessments of other infectious diseases. The questionnaire was subjected to psychometric evaluation before the data collection and found to be a valid and reliable measure. The Cronbach’s alpha coefficients for the dimensions ranged from 0.69 to 0.79, which indicated acceptable internal consistency for the questionnaire. In addition, the stability of the questionnaire as assessed by Intraclass Correlation Coefficient (ICC) showed satisfactory results (ICC ranged from 0.71 to 0.80) among (*n* = 195) same. The questionnaire was pilot tested with a sample of 420 respondents. The final questionnaire consisted of 29 items tapping into three pre-defined dimensions:

- 1. Efficacy (perceived self-efficacy and perceived response efficacy): This dimension included 13 items.
- 1.1 The perceived self-efficacy included six items measuring participants’ beliefs about their ability to perform the recommended responses to the COVID-19. A sample item reads as follows: “I am able to use a mask in closed places with more than two people.”
- 1.2 The response efficacy included seven items measuring participants’ beliefs about the effectiveness of the recommended preventative responses to the COVID-19. A sample items reads as follows: “I believe that by disinfecting surfaces, I am less likely to get coronavirus.”
- 2. Defensive response (denial, reactance and avoidance): This dimension included eight items measuring people’s beliefs about their perception of the risk of COVID-19.
- 2.1 A sample item for denial: “I think sickness or death is in God’s hands and following the precautionary measures is not so important”.
- 2.2 A sample item for reactance: “I believe that health staffs significantly exaggerate about the disease”.
- 2.3 A sample item for avoidance: “When TV or radio talks about coronavirus, I flip the channel”.
- 3. Perceived threats (susceptibility and severity). This dimension included eight items measuring people’s beliefs about the magnitude of the COVID-19 and about their risk of experiencing the disease.
- 3.1 A sample item for susceptibility: “I am at risk for getting the coronavirus”.
- 3.2. A sample item for severity: “The coronavirus is a lethal threat”.

Each item was rated on a 5-point Likert scale (1 = strongly disagree, 2 = disagree, 3 = neutral, 4 = agree, 5 = strongly agree) giving an overall row score ranging from 29 to 145. The rating for items belonging to defensive response were recoded so that to keep the direction of scoring as same as the other two dimensions (Additional file 1). Also the following demographic data were collected for all participants: age, gender, education, marital status, economic status, history of coronavirus, family history of coronavirus, having chronic diseases, and living condition (urban vs. rural).

### Scoring

1. Risk perception: Using the following simple linear transformation [Row score – the lowest possible raw score/highest possible raw score – the lowest possible raw score] × 100, the row scores were converted into a score of 0 to 100 where lower scores indicated lower risk perception and the higher scores indicated higher risk perception.

2. Danger control and fear control: Consistent with previous studies [9], we subtracted the perceived threat score from the perceived efficacy score (self- plus response-efficacy divided by two), resulting in a discriminating value. The discriminating value could be either positive or negative. A positive value meant that a person was engaging in danger control processes because their perceived efficacy was stronger than their threat perceptions. In other words, a person was likely to engage in some level of protective behaviors with regard to the specific health threat. A negative value meant that a person was engaging in fear control processes because their threat perceptions were stronger than their perceptions of efficacy. In these cases, a person was likely to engage in fear control processes and was probably not protecting himself or herself against the specific health threat.

### Statistical analysis

Statistical analyses were performed using the Statistical Package for Social Science, Version 18, for Windows (SPSS Inc., Chicago, IL, USA). The normality of the data was analyzed using a Kolmogorov-Smirnov test and the normal distribution of data was confirmed. The characteristics of the participants were summarized as numbers, percentages, or means with standard deviations, where appropriate. Analysis of variance (ANOVA), t-tests, and chi-square were used to compare the study sub-groups. In all tests, a value of *p* < 0.05 was considered statistically significant.

## Results

### Demographic characteristics

Table 1 shows the sociodemographic characteristics of the respondents (1933 male, and 1794 female). The mean age of the respondents was 37.0 (SD = 11.1) years. More than half of all participants (52%) were married. In all 40 respondents (1.1%) reported that they had coronavirus and 69 (1.9%) stated that they had a family member with a confirmed case of the disease. The majority of participants (96.1%) lived in urban areas.

**Table 1:** The characteristics of the study sample (*N* = 3727)

	Male (= 1933)	Female (***n*** = 1794)	Total
	No. (%)	No. (%)	No. (%)
**Age groups**			
15–29	376 (19.5)	489 (27.3)	865 (23.2)
30–44	1051 (54.4)	944 (52.6)	1995 (53.5)
45–59	419 (21.7)	315 (17.6)	734 (19.7)
60+	87 (4.5)	46 (2.6)	133 (3.6)
**Educational status**			
Primary	99 (5.1)	103 (5.7)	202 (5.4)
Secondary	458 (23.7)	465 (25.9)	923 (24.8)
Higher	1376 (71.2)	1226 (68.3)	2602 (69.8)
**Marital status**			
Single	439 (49.8)	442 (50.2)	881 (23.6)
Married	1483 (52.7)	1329 (47.3)	2812 (75.4)
Widowed/divorced	11 (0.6)	23 (1.3)	34 (0.9)
**Economic status**			
Good	378 (19.6)	547 (30.5)	925 (24.8)
Intermediate	1084 (56.1)	1049 (58.5)	2153 (57.2)
Poor	471 (24.4)	198 (11.0)	669 (17.9)
**History of coronavirus**			
Yes	16(0.9)	21 (1.1)	37 (1.0)
No	1778 (99.1)	1912 (98.9)	3690 (99.0)
**History of coronavirus in family members**			
Yes	33 (1.7)	36 (2.0)	69 (1.9)
No	1758 (98.0)	1900 (98.3)	3658 (98.1)
**Having hypertension**			
Yes	171 (8.8)	116 (6.5)	287 (7.7)
No	1762 (91.2)	1678 (93.5)	3440 (92.3)
**Having diabetes**			
Yes	98 (5.1)	51 (2.8)	149 (4.0)
No	1835 (97.2)	1743 (94.9)	3578 (96.0)
**Having respiratory diseases**			
Yes	65 (3.4)	45 (2.5)	110 (3.0)
No	1868 (96.6)	1749 (97.5)	3617 (97.0)
**Having cardiovascular diseases**			
Yes	76 (3.9)	42 (2.3)	118 (3.2)
No	1857 (96.1)	1752 (97.7)	3609 (96.8)
**Having other diseases**			
Yes	177 (9.1)	196 (10.8)	373(9.9)
No	1760 (91.1)	1600 (89.2)	3360 (90.2)
**Living condition**			
Urban	1862 (96.4)	1717 (95.7)	3579 (96.1)
Rural	71 (3.6)	77 (4.3)	148 (3.9)

### Perceived risk of the coronavirus disease

The perceived risk for all participants based on sociodemographic characteristics are shown in Table 2. The results by age revealed that as age increased, the significant progressive increase in perceived self-efficacy, avoidance response and perceived susceptibility scores were observed. This applied particularly for participants aged 60 and over. There were no statistically significant differences between age groups for response efficacy, denial, reactance, and severity scores. Women had higher but not significant scores than men for self-efficacy and response efficacy. Men had higher perceived susceptibility scores for the COVID-19 than women. The average scores across all dimensions showed significant increases as education levels increased, as well as for participants who were married or had good economic status. Perceived risk scores showed that participants with confirmed coronavirus cases had significantly higher scores except for the perceived susceptibility scores. Participants without any family history of coronavirus had higher perceived scores for response efficacy and self-efficacy. We found significantly higher scores for perceived risk among respondents with no chronic diseases (Table 3).

**Table 2:** Risk perception based on the EPPM by demographic characteristic (*N* = 3727)

	Efficacy		Defensive response			Threats	
	Self-efficacy	Response efficacy	Denial	Reactance	Avoidance	Susceptibility	Severity
	Mean (SD)	Mean (SD)	Mean (SD)	Mean (SD)	Mean (SD)	Mean (SD)	Mean (SD)
**Age (years)**							
15–29	65.6 (17.2)	84.0 (11.3)	23.2 (18.9)	19.8 (20.2)	31.1 (27.2)	65.5 (19.6)	76.4 (14.5)
30–44	65.6 (17.2)	83.5 (11.1)	22.0 (16.0)	19.2 (17.9)	26.6 (22.3)	69.1 (17.7)	77.1 (13.4)
45–59	68.3 (16.5)	84.2 (10.9)	21.3 (16.2)	18.9 (17.2)	24.3 (20.5)	67.7 (16.8)	77.0 (13.3)
60+	69.4 (16.9)	83.7 (11.0)	23.0 (17.5)	22.0 (17.6)	24.1 (16.9)	68.8 (16.3)	76.0 (14.0)
*P*-value	<.0001	0.473	0.134	0.309	<.0001	<.0001	0.539
**Gender**							
Female	67.6 (17.4)	85.0 (10.7)	22.0 (16.5)	17.5 (17.0)	26.0 (23.0)	66.1 (19.1)	76.9 (13.8)
Male	66.4 (16.8)	82.7 (11.3)	22.3 (17.3)	21.1 (19.4)	28.0 (23.4)	69.3 (17.2)	76.9 (14.1)
*P*-value	0.055	0.087	0.366	0.001	0.015	<.0001	0.56
**Educational status**							
Primary	68.1 (18.3)	81.7 (13.3)	29.2 (21.7)	25.8 (22.6)	31.9 (27.4)	59.2 (21.0)	74.3 (15.5)
Secondary	68.7 (16.5)	83.5 (10.9)	24.8 (18.0)	20.5 (19.0)	28.0 (24.1)	64.5 (19.1)	76.0 (13.8)
Higher	66.3 (17.2)	84.0 (10.9)	20.7 (15.9)	18.4 (17.6)	27.2 (23.2)	69.6 (17.3)	77.4 (13.9)
*P*-value	0.001	0.011	<.0001	<.0001	0.002	<.0001	0.001
**Marital status**							
Single	68.3 (16.9)	83.8 (10.9)	23.3 (18.4)	20.0 (20.1)	30.7 (25.9)	65.2 (20.2)	76.4 (14.3)
Married	66.6 (17.2)	83.9 (11.0)	21.7 (16.4)	19.1 (17.7)	25.9 (22.2)	68.6 (17.4)	77.1 (13.8)
Widowed/Divorced	63.3 (13.5)	78.5 (13.8)	26.9 (22.6)	21.5 (20.4)	32.7 (24.4)	62.7 (20.0)	74.4 (14.8)
*P*-value	0.024	0.021	0.017	0.355	<.0001	<.0001	0.224
**Economic status**							
Poor	63.1 (18.0)	81.6 (13.0)	23.9 (16.9)	20.8 (18.7)	27.8 (24.4)	67.5 (18.6)	77.9 (14.1)
Intermediate	66.4 (16.5)	83.6 (10.3)	22.0 (16.4)	19.6 (18.2)	27.1 (22.7)	67.9 (17.7)	76.5 (13.6)
Good	71.1 (17.0)	85.9 (10.7)	21.2 (18.1)	17.7 (17.7)	26.6 (23.2)	67.6 (19.0)	77.2 (14.7)
*P*-value	<.0001	<.0001	0.007	0.003	0.603	0.841	0.050
**Living condition**							
Urban	67.0 (17.1)	83.7 (11.1)	22.1 (16.8)	19.5 (18.3)	27.3 (23.2)	67.7 (18.2)	77.0 (14.0)
Rural	67.8 (18.5)	84.7 (10.1)	22.1 (19.5)	16.5 (18.3)	23.1 (22.3)	68.1 (18.2)	74.8 (13.2)
*P*-value	.051	.55	1.00	.051	0.033	0.817	0.066

**Table 3:** Risk perception based on the EPPM by disease status (*N* = 3727)

	Efficacy		Defensive response			Threats	
	Self-efficacy	Response efficacy	Denial	Reactance	Avoidance	Susceptibility	Severity
	Mean (SD	Mean (SD	Mean (SD	Mean (SD	Mean (SD	Mean (SD)	Mean (SD)
**History of coronavirus**							
Yes	57.7 (21.9)	74.9 (23.5)	32.8 (23.7)	30.1 (26.8)	41.5 (28.7)	70.0 (22.5)	67.4 (21.3)
No	67.1 (17.7)	83.9 (10.8)	22.0 (16.8)	19.3 (18.2)	27.0 (23.1)	67.7 (18.2)	77.9 (13.9)
*P*-value	0.036	< 0.0001	< 0.0001	0.009	< 0.0001	0.442	0.006
**History of coronavirus in family members**							
Yes	64.5 (20.9)	81.5 (18.9)	24.5 (19.7)	17.7 (20.5)	30.0 (27.8)	68.6 (22.1)	72.9 (17.4)
No	67.0 (17.0)	83.8 (10.8)	22.1 (16.9)	19.4 (18.3)	27.1 (23.1)	67.7 (18.1)	77.0 (13.9)
*P*-value	0.018	< 0.0001	0.25	0.32	0.45	0.031	0.142
**Frequency of chronic diseases**							
0	67.5 (16.9)	84.1 (10.8)	21.8 (16.9)	19.3 (18.3)	27.1 (23.5)	69.3 (18.6)	76.5 (13.9)
1	65.2 (17.7)	83.1 (11.5)	23.0 (17.0)	19.3 (18.1)	27.3 (22.4)	67.6 (16.7)	78.4 (14.0)
2	65.7 (16.4)	82.8 (11.6)	26.1 (19.0)	22.7 (20.4)	27.9 (21.0)	69.0 (15.7)	77.2 (12.8)
3 and more	60.0 (21.3)	77.6 (18.3)	20.8 (14.6)	17.2 (16.2)	20.0 (22.1)	66.6 (22.2)	74.8 (19.7)
*P*-value	0.001	0.002	0.026	0.224	0.390	0.021	0.016

### Danger control and fear control

Table 4 shows the discriminating values indicating danger control and fear control scores based on different sociodemographic characteristics. A total of 56.4% of participants were engaging in danger control processes and 43.6% in fear control processes. The respondents in former group were more likely to engage in preventive behaviors while those in the latter group were more likely to delay recommended responses for preventing themselves from the COVID-19. There were significant differences in danger and fear control scores by age, gender, education, economic status, and having chronic diseases.

**Table 4:** Danger control and fear control by demographic characteristics of the study sample (*N* = 3727)

	Danger control	Fear control	***P***-value
	No. (%)	No. (%)	
**Age**			< 0.0001
15–29	531 (25.3)	334 (20.5)	
30–44	1061 (50.5)	934 (57.4)	
45–59	434 (20.7)	300 (18.5)	
60+	75 (3.6)	58 (3.6)	
**Gender**			< 0.0001
Female	1079 (51.4)	715 (44.0)	
Male	1022 (48.6)	911 (56.0)	
**Educational status**			< 0.0001
Primary	142 (6.8)	60 (3.7)	
Secondary	595 (28.3)	328 (20.2)	
Higher	1238 (76.1)	1364 (64.9)	
**Marital status**			0.133
Single	519 (24.7)	362 (22.3)	
Married	1566 (74.5)	1246 (76.6)	
Widowed/Divorced	16 (0.8)	18 (1.1)	
**Economic status**			<.0001
Good	582 (27.7)	343 (21.1)	
Not good, not bad	1194 (56.8)	939 (57.7)	
Poor	325 (15.5)	344 (21.2)	
**History of coronavirus**			0.132
Yes	17 (0.8)	20 (1.2)	
No	2084 (99.2)	1606 (98.8)	
**History of coronavirus in a family member**			0.141
Yes	34 (49.6)	35 (2.2)	
No	2067 (98.4)	1591 (97.8)	
**Frequency of chronic diseases**			< 0.0001
0	1677 (79.8)	1202 (73.9)	
1	346 (16.5)	357 (22.0)	
2	66 (3.1)	49 (3.0)	
3 and more	12 (0.6)	18 (1.1)	
**Living condition**			0.555
Urban	2014 (95.9)	1565 (96.2)	
Rural	87 (4.1)	61 (3.8)	
**All**	2101 (56.4)	1626 (43.6)	

## Discussion

This EPPM-based study was conducted to assess the risk perceptions, overall perceived danger and fear control processes among Iranian people during the early stages of the COVID-19 pandemic. The study provides a timely assessment and initial evidence related to the risk perceptions and psychological responses of more than 3727 individuals across the country who took part in the study. In this study, the risk perception was evaluated through three dimensions of the EPPM including efficacy (self-efficacy and response efficacy), defensive response (denial, reactance, and avoidance), and threat (susceptibility and severity).

The study results showed that 56.4% of respondents were motivated by danger control responses and 43.6% by fear control responses. This indicates that more than half of all participants had high perceived efficacy (i.e., self-efficacy and response efficacy). According to the EPPM, two cognitive appraisals might initiate after a person learns about a health risk: one related to the threat it poses and a second related to the efficacy to follow the recommended responses. When the threat of COVID-19 is perceived to be more significant and efficacy is low, people are usually act to protect themselves from the fear rather than the danger itself (fear control process). Conversely when perceived efficacy is significantly high, even if the perceived treat would be high, people usually are motivated to protect themselves from the danger and could manage the threat (danger control process) [13].

Self-efficacy scores were significantly higher among participants who were older, female, single, lived in rural areas, or had good economic status. Self-efficacy is a positive mental state that is part of the cognitive appraisal process reducing stress and tension [14]. A current study from China showed an association between self-efficacy and social support among patients who had been treated for coronavirus [15]. This is inconsistent with our results, where it was found that participants with a history of coronavirus had lower self-efficacy scores. Respondents who had a family member with coronavirus and those with three or more comorbidities had lower self-efficacy scores for controlling COVID-19. The results showed that respondents with high self-efficacy were better able to control their emotions. Self-efficacy contributes to preventive behavior and the ability to conduct healthy behavior [16]. In this regard, a study by Liao et al. showed that self-efficacy was significantly associated with trust in government and media information on pandemic of A/H1N1 influenza [17]. We found that efficacy was significantly higher among respondents who were well-educated and had good economic status. It seems that these individuals believed that they can carry out the recommended responses to protect themselves from the COVID-19.

Individuals usually use psychological defense strategies to control their fears. These strategies include denial, avoidance, and reactance. Our results showed that higher defensive response scores correlated with better responses from participants. Defensive avoidance occurs when individuals block out feelings and thoughts about a threat or ignore further information about it, for example, switching the television channel or skipping COVID-19-related news. People in younger age groups had lower reactance scores and lower self-efficacy scores, indicating that younger people tended to take more risks and ignore health recommendations [18].

The results also showed that respondents who were male, older, well-educated, and married had significantly higher perceptions of susceptibility. In fact, these individuals were simply thinking about the threat of the COVID-19 and believed that the threat was relevant to them. According to the WHO, older people are at higher risk of contracting COVID-19 [19]. The Iranian health care system and media provided significant coverage of the COVID-19 pandemic, recommending that all people, especially older people take good care of themselves. This likely resulted in older people learning that they were more susceptible to the disease. However, some studies reported that after the initial stage of a pandemic the media attention to the topic declined and perceived susceptibility and severity declined accordingly [20–22].

### Strengths and limitations

This study benefited from a relatively good sample size and using a theory based questionnaire for data collection was an advantage. The greatest strength of this study was its format. The online method allowed for the timely collection of information from a wide range of community groups. Since the pandemic feature of the COVID-19 made other data collection methods unsafe and difficult for both the researchers and the study participants, the online sampling method was particularly convenient. However, because of the online nature of the study, we were unable to reach people who did not have access to the Internet or online applications. In addition, it is necessary to mention that online survey during the early stage of a pandemic was relatively new and therefore although some people received the invitation to participate, they did not attend to respond to the questionnaire. In fact, we could not identify none responders. Furthermore, most participants were relatively well educated. Thus the findings might not be generalized to all population. Finally, the present study did not introduce the cut-off values for the three dimensions of the questionnaire. Perhaps the future studies could indicate these values for screening proposes.

## Conclusion

More than half of all participants motivated by danger control. This indicated that more than half of participants had high perceived efficacy (i.e., self-efficacy and response efficacy). The results suggest that the risk perception of COVID-19 differs by socio economic and demographic characteristics. Indeed, the knowledge provided by the current study will likely contribute to the effectiveness of COVID-19 control and prevention measures.

## Supplementary information

**Additional file 1.** The Risk-Percept COVID-19 Questionnaire. The file contains a 29-items questionnaire which was specially developed for the study.

## Data Availability

The questionnaires and datasets generated and/or analyzed during the current study are available from the corresponding authors on reasonable request.

## Abbreviations

WHO: World Health Organization
EPPM: Extended parallel process model
ANOVA: Analysis of variance
COVID-19: Coronavirus disease 2019
