# Supplementary material for: Risk perception related to COVID-19 among the Iranian general population: an application of the extended parallel process model
Source: BMC Public Health. 2020 Oct 19;20:1571. doi: 10.1186/s12889-020-09681-7 (PMC7570396; doi:10.1186/s12889-020-09681-7)
Supplement: Supplementary file 1 — Additional file 1. The Risk-Percept COVID-19 Questionnaire. The file contains a 29-items questionnaire which was specially developed for the study. [file 12889_2020_9681_MOESM1_ESM.docx]

**Supplemental Materials**

**For**

**Transcriptional expression changes during compensatory plasticity in the central nervous system of the adult cricket *Gryllus bimaculatus***

**II. Escape system plasticity in the terminal ganglia**

Meera P. Prasad, Donald K. Detchou, Felicia Wang, Lisa L. Ledwidge, Sarah E. Kingston^^•^, Hadley W. Horch*,

Department of Biology, Bowdoin College, 6500 College Station, Brunswick, Maine 04011 USA.

Current address:

^School of Marine Sciences and Darling Marine Center, University of Maine, 193 Clarks Cove Rd., Walpole, ME 04573 USA;

^•^University of California Santa Cruz, Ecology and Evolutionary Biology Department and UC Natural Reserves, 1156 High St, Santa Cruz, CA 95064 USA

*Correspondence to:

Dr. Hadley Wilson Horch, Department of Biology, Bowdoin College 6500 College Station, Brunswick ME 04011 USA. Phone: 207-798-4128; FAX: 207-725-3405; Email: hhorch@bowdoin.edu

**Supplemental Materials:**

Supplemental File 1: Table 1, Counts matrix for all samples.

Supplemental File 2: Table 2, Transcripts identified as significantly upregulated at or below p = 0.05, 1 day post cercal removal by DESeq2. Column headings are as follows: qaccver (Query accesion.version) saccver (Subject accession.version), qstart (start of alignment in query), qend (end of alignment in query), sstart (start of alignment in subject), send (end of alignment in subject), bitscore, qframe (Query frame), evalue (expect value), sframe (Subject frame), ssciname (Subject Scientific Name), scomname (Subject Common Name), salltitles (All Subject Titles).

Supplemental File 3: Table 3, Transcripts identified as significantly upregulated at or below p = 0.05, 3 days post cercal removal by DESeq2. Column headings are as follows: qaccver (Query accesion.version) saccver (Subject accession.version), qstart (start of alignment in query), qend (end of alignment in query), sstart (start of alignment in subject), send (end of alignment in subject), bitscore, qframe (Query frame), evalue (expect value), sframe (Subject frame), ssciname (Subject Scientific Name), scomname (Subject Common Name), salltitles (All Subject Titles).

Supplemental File 4: Table 4, Transcripts identified as significantly upregulated at or below p = 0.05, 7 days post cercal removal by DESeq2. Column headings are as follows: qaccver (Query accesion.version) saccver (Subject accession.version), qstart (start of alignment in query), qend (end of alignment in query), sstart (start of alignment in subject), send (end of alignment in subject), bitscore, qframe (Query frame), evalue (expect value), sframe (Subject frame), ssciname (Subject Scientific Name), scomname (Subject Common Name), salltitles (All Subject Titles).

Supplemental File 5: Table 5, Transcripts identified as significantly downregulated at or below p = 0.05, 1 day post cercal removal by DESeq2. Column headings are as follows: qaccver (Query accesion.version) saccver (Subject accession.version), qstart (start of alignment in query), qend (end of alignment in query), sstart (start of alignment in subject), send (end of alignment in subject), bitscore, qframe (Query frame), evalue (expect value), sframe (Subject frame), ssciname (Subject Scientific Name), scomname (Subject Common Name), salltitles (All Subject Titles).

Supplemental File 6: Table 6, Transcripts identified as significantly downregulated at or below p = 0.05, 3 days post cercal removal by DESeq2. Column headings are as follows: qaccver (Query accesion.version) saccver (Subject accession.version), qstart (start of alignment in query), qend (end of alignment in query), sstart (start of alignment in subject), send (end of alignment in subject), bitscore, qframe (Query frame), evalue (expect value), sframe (Subject frame), ssciname (Subject Scientific Name), scomname (Subject Common Name), salltitles (All Subject Titles).

Supplemental File 7: Table 7, Transcripts identified as significantly downregulated at or below p = 0.05, 7 days post cercal removal by DESeq2. Column headings are as follows: qaccver (Query accesion.version) saccver (Subject accession.version), qstart (start of alignment in query), qend (end of alignment in query), sstart (start of alignment in subject), send (end of alignment in subject), bitscore, qframe (Query frame), evalue (expect value), sframe (Subject frame), ssciname (Subject Scientific Name), scomname (Subject Common Name), salltitles (All Subject Titles).

Supplemental File 8: Table 8, Transcripts identified as significantly upregulated at or below p = 0.05, 1 day post cercal removal by EdgeR. Column headings are as follows: qaccver (Query accesion.version) saccver (Subject accession.version), qstart (start of alignment in query), qend (end of alignment in query), sstart (start of alignment in subject), send (end of alignment in subject), bitscore, qframe (Query frame), evalue (expect value), sframe (Subject frame), ssciname (Subject Scientific Name), scomname (Subject Common Name), salltitles (All Subject Titles).

Supplemental File 9: Table 9, Transcripts identified as significantly upregulated at or below p = 0.05, 3 days post cercal removal by EdgeR. Column headings are as follows: qaccver (Query accesion.version) saccver (Subject accession.version), qstart (start of alignment in query), qend (end of alignment in query), sstart (start of alignment in subject), send (end of alignment in subject), bitscore, qframe (Query frame), evalue (expect value), sframe (Subject frame), ssciname (Subject Scientific Name), scomname (Subject Common Name), salltitles (All Subject Titles).

Supplemental File 10: Table 10, Transcripts identified as significantly upregulated at or below p = 0.05, 7 days post cercal removal by EdgeR. Column headings are as follows: qaccver (Query accesion.version) saccver (Subject accession.version), qstart (start of alignment in query), qend (end of alignment in query), sstart (start of alignment in subject), send (end of alignment in subject), bitscore, qframe (Query frame), evalue (expect value), sframe (Subject frame), ssciname (Subject Scientific Name), scomname (Subject Common Name), salltitles (All Subject Titles).

Supplemental File 11: Table 11, Transcripts identified as significantly downregulated at or below p = 0.05, 1 day post cercal removal by EdgeR. Column headings are as follows: qaccver (Query accesion.version) saccver (Subject accession.version), qstart (start of alignment in query), qend (end of alignment in query), sstart (start of alignment in subject), send (end of alignment in subject), bitscore, qframe (Query frame), evalue (expect value), sframe (Subject frame), ssciname (Subject Scientific Name), scomname (Subject Common Name), salltitles (All Subject Titles).

Supplemental File 12: Table 12, Transcripts identified as significantly downregulated at or below p = 0.05, 3 days post cercal removal by EdgeR. Column headings are as follows: qaccver (Query accesion.version) saccver (Subject accession.version), qstart (start of alignment in query), qend (end of alignment in query), sstart (start of alignment in subject), send (end of alignment in subject), bitscore, qframe (Query frame), evalue (expect value), sframe (Subject frame), ssciname (Subject Scientific Name), scomname (Subject Common Name), salltitles (All Subject Titles).

Supplemental File 13: Table 13, Transcripts identified as significantly downregulated at or below p = 0.05, 7 days post cercal removal by EdgeR. Column headings are as follows: qaccver (Query accesion.version) saccver (Subject accession.version), qstart (start of alignment in query), qend (end of alignment in query), sstart (start of alignment in subject), send (end of alignment in subject), bitscore, qframe (Query frame), evalue (expect value), sframe (Subject frame), ssciname (Subject Scientific Name), scomname (Subject Common Name), salltitles (All Subject Titles).

Supplemental File 14: Table 14, Blast2GO results for candidates identified as upregulated in the TAG by both EdgeR and DESeq2 1 day after cercal removal.

Supplemental File 15: Table 15, Blast2GO results for candidates identified as upregulated in the TAG by both EdgeR and DESeq2 3 days after cercal removal.

Supplemental File 16: Table 16, Blast2GO results for candidates identified as upregulated in the TAG by both EdgeR and DESeq2 7 days after cercal removal.

Supplemental File 17: Table 14, Blast2GO results for candidates identified as downregulated in the TAG by both EdgeR and DESeq2 1 day after cercal removal.

Supplemental File 18: Table 15, Blast2GO results for candidates identified as downregulated in the TAG by both EdgeR and DESeq2 3 days after cercal removal.

Supplemental File 19: Table 16, Blast2GO results for candidates identified as downregulated in the TAG by both EdgeR and DESeq2 7 days after cercal removal.

Supplemental File 20: Table 20, Swiss-Prot matches for candidates identified as upregulated in the TAG by both EdgeR and DESeq2 1 day after cercal removal.

Supplemental File 21: Table 21, Swiss-Prot matches for candidates identified as upregulated in the TAG by both EdgeR and DESeq2 3 days after cercal removal.

Supplemental File 22: Table 22, Swiss-Prot matches for candidates identified as upregulated in the TAG by both EdgeR and DESeq2 7 days after cercal removal.

Supplemental File 23: Table 23, Swiss-Prot matches for candidates identified as downregulated in the TAG by both EdgeR and DESeq2 1 day after cercal removal.

Supplemental File 24: Table 24, Swiss-Prot matches for candidates identified as downregulated in the TAG by both EdgeR and DESeq2 3 days after cercal removal.

Supplemental File 25: Table 25, Swiss-Prot matches for candidates identified as downregulated in the TAG by both EdgeR and DESeq2 7 days after cercal removal.
